# Efficacy and Safety of a Home‐Use Light‐Emitting Diode Neck Device for Improvement in Neck Wrinkles: A Multicenter, Randomized, Double‐Blind, Sham Device, Superiority Verification, Confirmatory Clinical Trial

**DOI:** 10.1111/jocd.16599

**Published:** 2024-09-25

**Authors:** Seong Oh Park, Jiwoo Jang, Sang Hyun Park, Jae‐A Jung, Chihwan Cha, Bo Hyun Lee, Jung Kwon An

**Affiliations:** ^1^ Department of Plastic and Reconstructive Surgery Seoul National University Hospital, Seoul National University College of Medicine Seoul Korea; ^2^ Department of Plastic and Reconstructive Surgery Hanyang University College of Medicine Seoul Korea; ^3^ Department of Surgery Hanyang University College of Medicine Seoul Korea

**Keywords:** LED application, neck rejuvenation, neck wrinkles, safety testing, skin physiology

## Abstract

**Background:**

Light‐emitting diode (LED) light sources have become an increasingly popular choice for the treatment and rejuvenation of various dermatological conditions.

**Aims:**

This study aimed to evaluate the effects of neck rejuvenation, patient satisfaction, and the safety of LED application to the neck in an Asian population.

**Methods:**

This was a multicenter, randomized, double‐blind, sham device study. Seventy participants were enrolled in the study. The participants wore the home‐use LED neck device for 9 min a day, 5 times a week, for a total of 60 sessions. The Lemperle Wrinkle Scale (LWS) and Global Aesthetic Improvement Scale (GAIS) were used to evaluate the results of both investigators and participants. The thyroid gland was examined using ultrasonography to evaluate the safety of the investigational device.

**Results:**

The percentage of participants with improved LWS at Week 12 was significantly higher in the study group. Additionally, the percentage of participants with improved LWS was significantly higher in the study group at Weeks 8, 12, and 16. The LWS at Week 12 corrected with baseline values was found to be significantly different between the two groups. GAIS showed significant differences at 8, 12, and 16 weeks in the investigators' evaluation but not in the participants' evaluation. Repeated‐measures analysis of variance at Weeks 4, 8, 12, and 16 also confirmed a significant difference between the two groups only in investigator assessment. No significant thyroid‐related complications were observed.

**Conclusion:**

LED application to the neck may be considered a satisfactory and safe procedure for neck rejuvenation.

## 1 Introduction

Aging of neck skin is one of the earliest signs of aging. Aging necks exhibit undesired morphological changes, including wrinkles, pigmentation, telangiectasia, and loss of elasticity. While the face can be covered in many ways, including using makeup, the neck is often not covered, which is troublesome for many people [1]. The dermis and subcutaneous fat layers of neck skin comprise approximately two‐thirds of the skin on the face, and there are few sebaceous glands; therefore, it dries out easily, and there is less blood supply and distribution of skin appendages essential for regeneration. The neck is also prone to wrinkles because it has fewer muscles and is relatively mobile compared with the face. Wrinkles that appear in one's 20s are caused by atrophy of subcutaneous fat in the neck skin, which causes the subcutaneous tissue to lose its strength and become progressively deeper as one passes their 40s. Vertical wrinkles that form two straight lines as the platysma muscle, which is spread thinly like a butcher's block on both sides of the neck, continuously contract as one reaches their 40s and 50s [1].

Various rejuvenation modalities have been developed. The most widely used invasive method is neck‐lift surgery. It is important to release the skin from the retaining ligaments and redrape the skin with proper repositioning of soft tissue structures such as the subplatysmal fat, digastric muscle, and submandibular gland. Sometimes, minimally invasive surgical methods such as lipotectomy and platysmaplasty are performed using a submental liposuction or a submental‐only approach [2]. However, some patients are reluctant to undergo invasive surgical treatments.

Various nonsurgical treatments have also been used. These include botulinum toxin, injectable fillers, PDRN injections, ablative lasers, nonablative lasers, microfocused ultrasound, and high‐intensity focused ultrasound [3]. Ablative laser resurfacing is widely used with carbon dioxide, Er:YAG, or a combination of both. However, nonablative lasers are preferred because post‐treatment care after ablative laser treatment can be relatively uncomfortable for patients. Nonablative skin rejuvenation aims to rejuvenate the epidermis without damaging it. Typical examples include intense pulsed light and Nd:YAG lasers. Recently, microfocused ultrasound has been widely used, and it has shown significant effects on noninvasive skin tightening and lifting of the lower face and neck. The principle is that the temperature can be raised to 65° by transducing direct ultrasound energy to focal layers such as the dermis and superficial musculoaponeurotic system, causing tissue coagulation and collagen contraction [4].

In recent years, light‐emitting diode (LED) light sources have become increasingly popular for treating dermatological conditions [5]. Unlike high‐powered lasers, which focus on a localized area, LED light sources can effectively treat large areas of skin with an appropriate light output. The US Food and Drug Administration has cleared LED light sources for human use in the visible and near‐infrared regions of the spectrum because they emit specific wavelengths of light owing to their narrow wavelength bandwidth. This means that they do not emit harmful ultraviolet or unwanted infrared (IR) radiation, and their low energy does not damage skin tissue or eyes. The physical characteristics of LED light sources include a long lifespan, low power consumption, environmental friendliness, and small volume for easy space utilization [6].

This prospective multicenter randomized placebo‐controlled double‐blind superiority verification and confirmatory clinical trial aimed to compare and evaluate the efficacy and safety of a home‐use LED neck device for the improvement in neck wrinkles.

## 2 Materials and Methods

### 2.1 Study Participants

This study included Asians between 30 and 65 years of age with Fitzpatrick types II–V. Individuals who wanted to improve the appearance of neck wrinkles and scored between 2 and 4 on the Lemperle Wrinkle Scale (LWS) were included in the study. The LWS scoring was as follows: 0 = no wrinkles; 1 = barely perceptible wrinkle; 2 = shallow wrinkle; 3 = moderately deep wrinkle; 4 = deep wrinkle, well‐defined edges; and 5 = very deep wrinkle, redundant fold [7, 8]. The exclusion criteria were as follows: had undergone cosmetic treatments such as laser, light therapy, surgery, or filler treatments using collagen, hyaluronic acid fillers, or other materials in the neck area within the previous 6 months and radiation therapy to the neck area; a history of malignancy or connective tissue diseases and infections or abnormal skin lesion in the neck; used whitening agents such as hydroquinone and tranexamic acid, isotretinoin or retinoid, light‐sensitive medications, steroid preparations within the previous 6 months, anti‐wrinkle functional cosmetics containing retinol, retinyl palmitate, adenosine, and polyethoxylated retinamide within the previous 3 months or unable to stop using them; and pregnancy and lactation. This clinical trial was approved by the Institutional Review Board (IRB No. 2022‐12‐016). Written consent was provided by which the patients agreed to the use and analysis of their data. Two hospitals enrolled the participants, and each hospital ran the clinical trial independently.

### 2.2 Study Design

**FIGURE 1:**
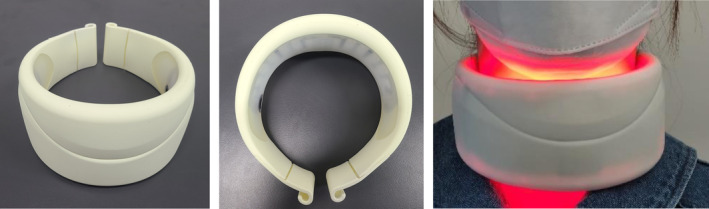
Home‐use LED neck device and the actual appearance of the application.

This work was supported by Y&J BIO (Hanam‐si, Gyeonggi‐do, Korea), who provided financial support for the study. This was a multicenter, randomized, double‐blind sham device study. Based on similar studies, the test for two proportions (Fisher's exact test) in the sample size calculation program PASS (NASS Statistical Software, Kaysville UT, a sample size calculation program) concluded that 70 participants were required considering a dropout rate of 20%. The participants wore a home‐use LED neck device (630 nm LED/850 nm IRED, Easy Claire Neck Solution, Y&J Bio) for 9 min a day, five times a week, for a total of 60 sessions (12 weeks, 540 min). The power intensity was 14 mW/cm^2^ with 630 nm LED and 8 mW/cm^2^ with 850 nm IRED. The maximum total irradiation energy was 784.08 J/cm^2^. The control device (sham device) had the same appearance and components, was not irradiated with IR (850 nm), was manufactured to irradiate only one‐tenth of the LED (630 nm), and was used in the same way as the test device; therefore, the test and control devices were indistinguishable (Figure 1). During the study, participants attended up to six visits, including a screening baseline visit immediately after the first application of the investigational device (4 weeks post‐baseline), 4 weeks after completion of application (8 weeks post‐baseline), 8 weeks (12 weeks post‐baseline), 12 weeks (16 weeks post‐baseline), and 4 weeks after the 12th week of application of investigational device for efficacy and safety assessments and safety follow‐up, for a total of 16 weeks.

### 2.3 Measurement

**FIGURE 2:**
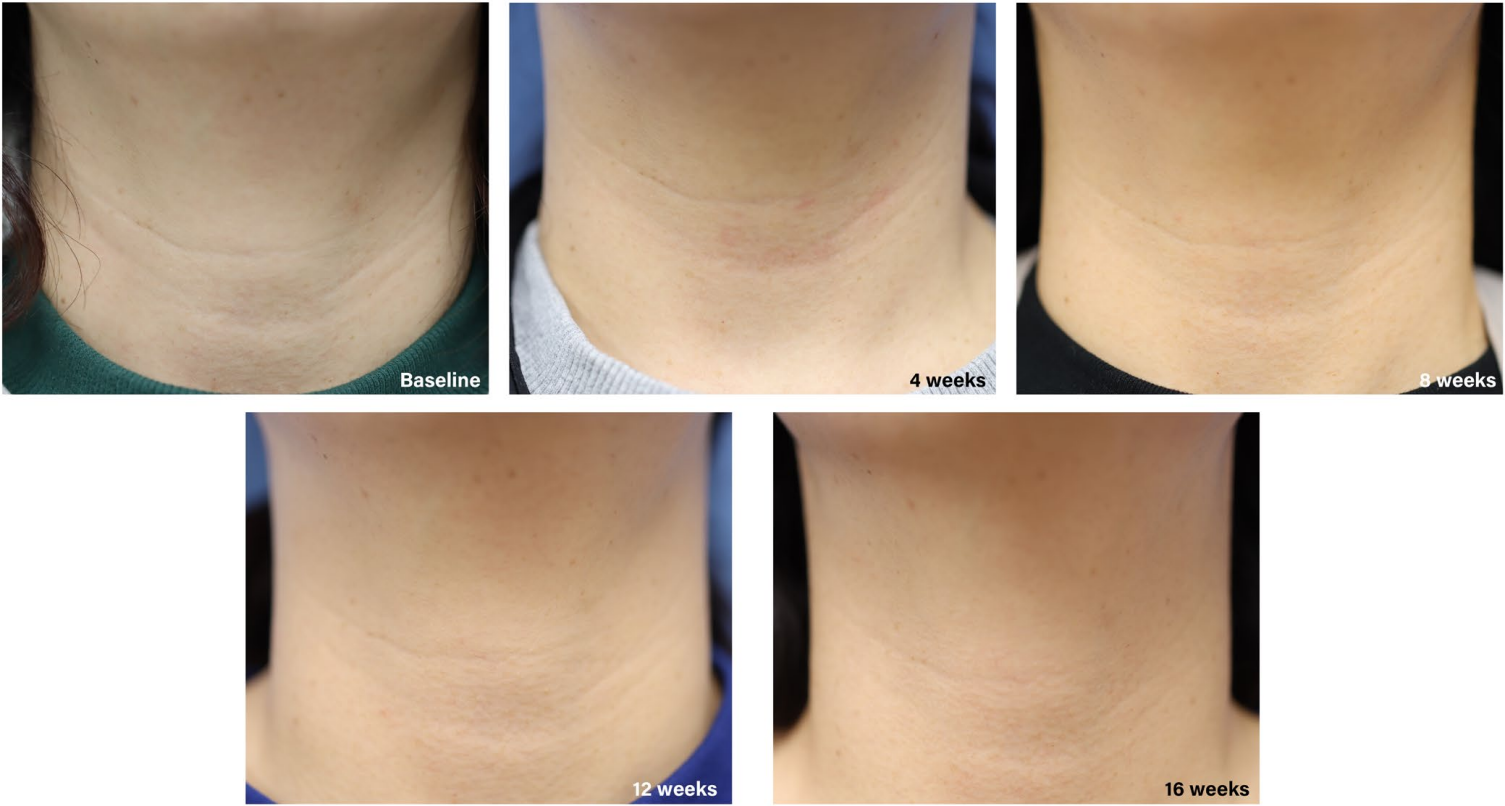
Clinical photographs showing an improvement in neck wrinkles after device application.

An evaluation was performed at each visit to assess wrinkle improvement. Photographs of the participants' necks taken at each time point were evaluated. Standardized photographs were taken uniformly using a Canon EOS 600D with an EF of 50 mm (f/1.8) (Canon Inc., Tokyo, Japan) in the same setting. Since the nature of neck wrinkles is such that leaning the face forward creates more wrinkles and leaning it back can stretch them out, all participants were photographed with their necks and spines in a straight line (Figure 2). The investigator evaluated the LWS by three blinded plastic surgeons by evaluating serial clinical photographs with random numbers. Global Aesthetic Improvement Scale (GAIS) assessments were performed at 4, 8, 12, and 16 weeks after baseline to evaluate the post‐treatment aesthetic improvement in neck wrinkles. The GAIS scale was as follows: Grade 1 = worse; Grade 2 = no change; Grade 3 = somewhat improved; Grade 4 = moderately improved; and Grade 5 = very much improved. The GAIS assessment was performed by the investigator, and participants were assessed by direct visual observation without the use of photographs.

### 2.4 Thyroid Exanimation

To evaluate the safety of the investigational medical device, the thyroid gland was examined using ultrasonography with a 5–12 MHz linear transducer. This was performed to check for changes in the size of preexisting thyroid nodules and identify new nodules. Thyroid evaluations were performed at baseline, 12 weeks after device application, and 4 weeks after application completion.

### 2.5 Statistical Analysis

Comparisons between the test and control groups were performed using Pearson's chi‐square test, Fisher's exact test, or the *Z* test at each time point. The significance of intragroup differences in the endpoints at each time point was tested using McNemar's test or McNemar's exact test. Continuous endpoints were compared between groups using an independent two‐sample *t*‐test or Mann–Whitney *U* test for normality at each time point and analysis of covariance using a general linear model corrected for baseline. Additionally, intragroup significance for changes from baseline in endpoints at each time point was tested using repeated‐measures analysis of variance (ANOVA) and a paired *t*‐test or Wilcoxon signed‐rank test for normality.

## 3 Results

### 3.1 Participants' Characteristics

Seventy (10 male and 60 female) of 77 participants completed the study protocol. The mean age of the study group was 44.94 (range 34–63), and that of the control group was 47.20 (range 31–63). There were no significant underlying conditions in either group, with high frequencies of dyslipidemia and hypertension, and no significant differences between the groups. The baseline LWS score was significantly higher in the study group (3.20 vs. 2.54, *p* = 0.009) (Table 1).

**TABLE 1:** Patients characteristics and primary outcome.

Characteristics	Study group (*n* = 35)	Control group (*n* = 35)	*p*
	*n* (%)	*n* (%)	
Sex			
Male	5 (14.3)	5 (14.3)	1.000
Female	30 (85.7)	30 (85.7)	
Age	44.94 ± 8.41	47.20 ± 7.70	0.246
Underlying disorder			
Dyslipidemia	3 (8.6)	4 (11.4)	1
Hypertension	5 (14.3)	3 (8.6)	0.141
Baseline LWS	3.2 ± 1.08	2.54 ± 0.89	0.009
Primary outcome			
Worsened	1 (2.9)	4 (11.4)	< 0.001*
No change	7 (20)	28 (80)	
Improvement	27 (77.1)	3 (8.6)	

Abbreviation: LWS, Lemperle wrinkle scale.

* Pearson Chi‐square test.

### 3.2 Clinical Efficacy

#### 3.2.1 Primary Outcome

The proportion of participants with improved neck wrinkles, estimated by the investigators using the LWS score at 12 weeks after device application, was 27 (77.1%) in the test group (35 participants) and 3 (8.6%) in the control group (35 participants). Pearson's chi‐square test showed that the difference between the test and control groups was significant (*p* = 0.000), thus confirming the effectiveness of the personal combinatorial stimulator for the temporary improvement in neck wrinkles (Table 1).

#### 3.2.2 Secondary Outcome

##### 3.2.2.1 LWS Score by Investigators Over Time

**FIGURE 3:**
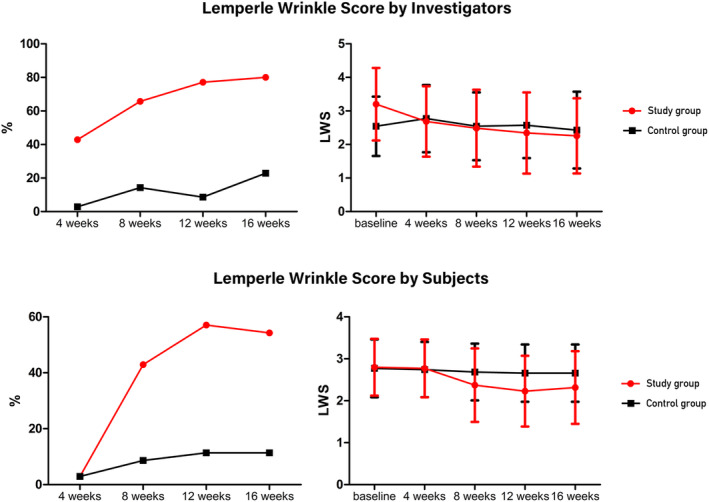
Lemperle Wrinkle Score (LWS) improvement rate and score change according to the investigators and subjects.

Comparison of the proportion of participants with improvement in neck wrinkles from baseline, as assessed by the investigators, revealed that the difference between the two groups was significant at 4, 8, 12, and 16 weeks after the application of the device (*p* < 0.001). McNemar's test was also performed to compare the intragroup differences at Week 4 versus Week 12, and no significant difference was observed in the treatment group; however, there was a significant difference in the control group (*p* = 0.070, 0.021). The intragroup comparison at Week 12 versus Week 16 was not significant in either the treatment (*p* = 0.375) or control (*p* = 0.250) group (Table 2, Figure 3). The LWS score at 12 weeks, corrected with the baseline value, was significant in both groups (*p* < 0.001). The change over time in both groups was not significant (*p* = 0.923). Additionally, an intragroup comparison from baseline to 12 weeks using Wilcoxon rank‐sum revealed a significant difference in the treatment group but not in the control group (*p* < 0.000 vs. *p* = 0.705) (Table 3, Figure 3).

**TABLE 2:** Proportion of LWS improvement from baseline by investigators and participants.

	Study group (*n* = 35)	Control group (*n* = 35)	*p*
	*n* (%)	*n* (%)	
By investigators			
4 weeks	15 (42.9)	1 (2.9)	< 0.001^a^
8 weeks	23 (65.7)	5 (14.3)	< 0.001^a^
12 weeks	27 (77.1)	3 (8.6)	< 0.001^a^
16 weeks	28 (80.0)	8 (22.9)	< 0.001^a^
*p*	0.070^b^/0.375^c^	0.021^b^/0.250^c^	
By participants			
4 weeks	1 (2.9)	1 (2.9)	1.000^a^
8 weeks	15 (42.9)	3 (8.6)	0.002^a^
12 weeks	20 (57.1)	4 (11.4)	< 0.001^a^
16 weeks	19 (54.3)	4 (11.4)	< 0.001^a^
*p*	< 0.001^b^/1.000^c^	0.250^b^/1.000^c^	

^a^ Pearson Chi‐square test.

^b^ 4 weeks versus 12 weeks McNemar test.

^c^ 12 weeks versus 16 weeks McNemar test.

**TABLE 3:** LWS value over time by investigators and participants.

	Study group (*n* = 35)		Control group (*n* = 35)		*p*
	Mean	SD	Mean	SD	
By investigators					
Baseline	3.2	1.08	2.54	0.89	0.009^a^
4 weeks	2.69	1.05	2.77	1	0.782^a^
8 weeks	2.49	1.15	2.54	1.01	0.695^a^
12 weeks	2.34	1.21	2.57	0.98	0.181^a^
16 weeks	2.26	1.12	2.43	1.14	0.439^a^
*p*	< 0.001^b^		0.705^b^		0.923^c^
By participants					
Baseline	2.8	0.68	2.77	0.69	0.846^a^
4 weeks	2.77	0.69	2.74	0.66	0.887^a^
8 weeks	2.37	0.88	2.69	0.68	0.152^a^
12 weeks	2.23	0.84	2.66	0.68	0.039^a^
16 weeks	2.31	0.87	2.66	0.68	0.099^a^
*p*	< 0.001^b^		0.046^b^		0.222^c^

^a^ Mann–Whitney *U* test.

^b^ Wilcoxon rank‐sum test.

^c^ Repeated ANOVA.

##### 3.2.2.2 LWS Score by Participants Over Time

The percentage improvement in the participants was compared between the two groups using Pearson's chi‐square test, and the difference was not significant at 4 weeks after the first application of the investigational device (*p* = 1.000). However, the difference between the two groups was significant at 8 weeks (*p* = 0.002, < 0.001, < 0.001). McNemar's test was performed to compare intragroup differences between Weeks 4 and 12, and a significant difference was found in the test group but not in the control group (*p* < 0.001 and *p* = 0.250, respectively). The intragroup comparison at Week 12 versus Week 16 was not significant in either group (*p* = 1.000 and 1.000, respectively) (Table 2, Figure 3); however, participant‐assessed LWS at Week 12, corrected for baseline, was compared using covariate analysis and found to be significantly different between the two groups (*p* < 0.001). However, the difference in the change in scores was not significant according to the repeated‐measures ANOVA (*p* = 0.222). In a simple comparison at each time point, a significant difference was observed only at Week 12 (*p* = 0.039). Wilcoxon rank‐sum test was also performed for intra‐arm comparisons from baseline to 12 weeks, and significant differences were observed between the arms (*p* < 0.001 and *p* = 0.046, respectively) (Table 3, Figure 3).

##### 3.2.2.3 GAIS

**FIGURE 4:**
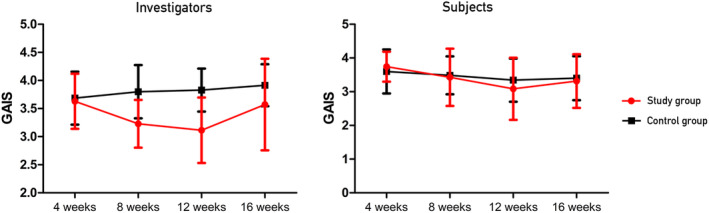
Change in the Global Aesthetic Improvement Scale (GAIS) according to the investigators and subjects.

GAIS scores were compared between the two groups using an analysis of covariance at Week 12, corrected for Week 4, and showed a significant difference between the two groups (*p* < 0.001). Additionally, repeated‐measures ANOVA was used to analyze the significant differences in the change in scores at Weeks 4, 8, 12, and 16 between the two groups (*p* < 0.000). The comparison of GAIS scores between the two groups was not significant at Week 4 (*p* = 0.617); however, after Week 8, the difference was significant (*p* < 0.001, *p* < 0.001, and *p* = 0.035, respectively). The Wilcoxon rank‐sum test for within‐group comparisons at Week 4 versus Week 12 showed a significant difference only in the test group (*p* = 0.000, 0.197) (Table 4, Figure 4).

**TABLE 4:** GAIS value over time by investigators and participants.

	Study group (*n* = 35)		Control group (*n* = 35)		*p*
	Mean	SD	Mean	SD	
By investigators					
4 weeks	3.63	0.49	3.69	0.47	0.617^a^
8 weeks	3.23	0.43	3.8	0.47	0.000^a^
12 weeks	3.11	0.58	3.83	0.38	0.000^a^
16 weeks	3.57	0.81	3.91	0.37	0.035^a^
*p*	< 0.001^b^		0.197^b^		0.000^c^
By participants					
4 weeks	3.74	0.44	3.6	0.65	0.400^a^
8 weeks	3.43	0.85	3.49	0.56	0.744^a^
12 weeks	3.09	0.92	3.34	0.64	0.327^a^
16 weeks	3.31	0.8	3.4	0.65	0.800^a^
*p*	< 0.001^b^		0.053^b^		0.629^c^

^a^ Mann–Whitney *U* test.

^b^ Wilcoxon rank‐sum test.

^c^ Repeated ANOVA.

The GAIS scores were evaluated using the same analytical method. A comparison of the GAIS scores at 12 weeks corrected for Week 4 between the two groups showed no significant difference between the two groups (*p* = 0.082). Repeated‐measures ANOVA revealed no significant difference between the two groups (*p* = 0.629). The GAIS scores between the treatment and control groups did not significantly differ between the two groups at Weeks 4, 8, 12, and 16 (*p* = 0.400, 0.744, 0.327, and 0.800, respectively). At Week 4 versus Week 12, intragroup comparisons revealed a significant difference only in the treatment group (*p* = 0.000 and *p* = 0.053, respectively) (Table 4, Figure 4).

### 3.3 Thyroid Examination

Changes in the sizes of the nodules and cysts found in the thyroid glands of all participants were evaluated. From baseline to 12 weeks, the size of thyroid nodules or cysts increased by 0.40 ± 2.36 mm in the treatment group, compared to 0.24 ± 3.03 mm in the control group. Four weeks after discontinuation, compared to Week 12, the size of thyroid nodules or cysts increased by 0.18 ± 1.01 mm in the treatment group and decreased by 0.14 ± 1.38 mm in the control group. None of the values were statistically significant (Table 5).

**TABLE 5:** Changes in the size of nodules and cysts found in the thyroid gland.

	Study group (*n* = 35)	Control group (*n* = 35)	*p*
	Size (mm)	Size (mm)	
Baseline	1.53 ± 3.03	1.68 ± 2.24	0.929^b^
12 weeks	1.93 ± 3.44	1.92 ± 2.95	
16 weeks	2.1 ± 4.01	1.92 ± 2.84	
*p*	0.440^a^	0.766^a^	

^a^ Wilcoxon rank‐sum test.

^b^ Repeated ANOVA.

Specifically, one participant in the study group showed a 15‐mm‐sized isoechoid nodule in Category 3 of the European Thyroid Imaging and Reporting Data System (EU‐TIRADS) at the initial examination. No significant changes were observed during follow‐up examinations. One participant in the study group and two in the control group showed a small thyroid nodule of < 5 mm that was not detected on initial examination but was not related to the device on ultrasound review. All lesions were Category 2 according to EU‐TIRADS, and no further testing was performed [9]. The overall results are presented in Table 6.

**TABLE 6:** Summary of findings.

Outcome	Study group	Control group
Primary outcome^a^	77.1% improved	8.6% improved
LWS by investigators	Significant improvement over time	No significant improvement
LWS by participants	Significant improvement over time	No significant improvement
GAIS by investigators	Significant improvement over time	No significant improvement
GAIS by participants	Significant improvement over time	No significant improvement
Thyroid Examination	No significant changes in thyroid nodule size	No significant changes in thyroid nodule size

Abbreviations: GAIS, Global Aesthetic Improvement Scale; LWS, Lemperle wrinkle scale.

^a^ Proportion of LWS improvement at 12 weeks.

## 4 Discussion

Research regarding LED therapy, a relatively new modality compared to laser therapy for skin diseases, began in the mid‐2000s. Since then, several companies have investigated LED therapy for various skin diseases and cosmetic purposes [10, 11]. In recent years, the need for in‐home devices or home care has risen as an increasing number of consumers are unable to receive regular medical care owing to time, location, or financial constraints. The range of home devices has expanded from LED devices to transdermal drug delivery systems [12].

In contrast to other nonablative rejuvenation methods, LED devices do not cause photothermal damage. Instead, they induce photobiomodulatory reactions. They also stimulate fibroblast proliferation, collagen synthesis, and extracellular matrix and growth factor production [13, 14, 15]. In an in vitro study, irradiation of human fibroblasts and skin samples with 640 nm plus 830 nm LED light at 0.5 mW/cm^2^ for 10 min increased *LOXL1*, *ELN*, *COL1A1*, and *COL3A1* gene expression and procollagen type I and elastin protein expression [16]. Several clinical studies have confirmed that the application of LED/IRED light at 600–660 nm and 800–860 nm has anti‐wrinkle and anti‐aging effects by stimulating cells in the dermis and epidermis. Additionally, 600–650 nm and 800–860 nm LEDs for collagen synthesis have been shown to increase the number of fibroblasts in the human dermis and the synthesis of collagen and elastin proteins [10, 13, 17].

To date, several studies on LED devices have focused on facial rejuvenation [18]. In one prospective single‐arm interventional study, a 630/850 nm home‐use neck LED device was applied to 30 participants for 16 weeks, and both investigator‐ and participant‐rated LWS and GAIS scores improved. There were no significant side effects on the thyroid and parathyroid glands [19]. The study showed similar results to those of the current study, indicating that a home‐use LED device is effective for neck rejuvenation. However, that study had only one arm, with no control group. To our knowledge, this is the first multicenter randomized clinical trial of neck rejuvenation using a home‐use LED device.

Neck rejuvenation studies have unique characteristics compared to facial rejuvenation studies. Although it is easier to observe changes in shallow wrinkles around the eyes, the neck area often has one to four relatively deep wrinkles, making marked changes harder to achieve with LED devices. The depth of wrinkles can vary with the individual's posture. Thus, posture was controlled as much as possible during participant evaluation and photography. Considering these aspects, we believe that the LWS and GAIS evaluated by the participants, although subjective, are crucial.

Additionally, since the device is applied to the neck, safety for the thyroid gland should be considered. Thyroid cancer is one of the most common cancers in East Asian countries [20]. The penetration depth of light in the human body at wavelengths of 630 and 850 nm is reported to be up to 2 and 3 mm, respectively, where most of the light is absorbed before reaching deeper tissues [21, 22]. Given the depth of penetration of red and infrared light into the body, its effect on the thyroid gland is expected to be minimal. No adverse events have been reported in clinical studies involving the thyroid gland and low‐level laser therapy [19]. No significant adverse effects were found in this study, as assessed by EU‐TIRAD.

Our study had some limitations. The evaluation of the results was conducted using only visual findings such as the LWS and GAIS. In previous studies, the physical properties of the skin were analyzed using equipment such as a visiometer, and melanin levels were also measured; however, this was omitted because the combined LED application of 630 and 850 nm provided sufficient positive evidence in previous studies [17]. Instead, we focused on thyroid assessment because the application site was the neck, and its safety has been demonstrated in other studies. The fact that baseline LWS was significantly different between the two groups could also be a source of bias. However, this was an unavoidable consequence of the randomization process, and we attempted to minimize its impact by correcting for the baseline LWS in the statistical analysis.

This clinical trial evaluated the neck rejuvenation effect of a home‐use LED neck device (Easy Claire Neck Solution) using LWS and GAIS. Based on the results, it was concluded that the Easy Claire Neck Solution had a positive effect on improving neck wrinkles without thyroid‐related complications.

## Author Contributions

Conception and design: Seong Oh Park, Jae‐A Jung, and Chihwan Cha. Acquisition of data: Seong Oh Park, Jiwoo Jang, Sang Hyun Park, Jae‐A Jung, Chihwan Cha, Bo Hyun Lee, and Jung Kwon An. Analysis and interpretation of data: Seong Oh Park, Jiwoo Jang, Jae‐A Jung, and Chihwan Cha. Writing or revision of the manuscript: Seong Oh Park, Jiwoo Jang, Sang Hyun Park, Bo Hyun Lee, and Jung Kwon An. Final approval: all authors.

## Conflicts of Interest

The authors declare no conflicts of interest.

## Data Availability

The authors have nothing to report.
